# Complete Genome Sequences of Nitrofurantoin-Sensitive and -Resistant *Escherichia coli* ST540 and ST2747 Strains

**DOI:** 10.1128/genomeA.00239-14

**Published:** 2014-04-10

**Authors:** Basil Britto Xavier, Jascha Vervoort, Andrew Stewardson, Niels Adriaenssens, Samuel Coenen, Stephan Harbarth, Herman Goossens, Surbhi Malhotra-Kumar

**Affiliations:** a Department of Medical Microbiology, Vaccine & Infectious Disease Institute, Universiteit Antwerpen, Antwerp, Belgium; b University of Geneva Hospitals and Faculty of Medicine, Geneva, Switzerland

## Abstract

Widespread multidrug resistance in *Escherichia coli* has necessitated the reintroduction of older antibiotics, such as nitrofurantoin. However, mechanisms by which resistance to nitrofurantoin emerges in *E. coli* are not well elucidated. Toward this aim, we sequenced two nitrofurantoin-sensitive *E. coli* sequence types (ST540 and ST2747) and their four nitrofurantoin-resistant derivatives generated *in vitro* under aerobic and anaerobic growth conditions.

## GENOME ANNOUNCEMENT

*Escherichia coli* is the most common cause of urinary tract infections (UTI) in the community (1). Multidrug resistance in *E. coli* has necessitated the reintroduction of older antibiotics, like nitrofurantoin. Nitrofurantoin acts by undergoing reduction by bacterial nitroreductases to generate toxic derivatives that attach to ribosomal proteins (2), causing defective transcription and translation in bacteria (3). First described in the 1970s, nitrofurantoin resistance in *E. coli* involves loss-of-function mutations in two genes encoding nitroreductases, *nfsA* and *nfsB* (4, 5). Although these mechanisms are well known, no previous studies have focused beyond the *nfs* genes.

We generated nitrofurantoin-resistant isolates *in vitro* under aerobic and anaerobic conditions from two nitrofurantoin-sensitive *E. coli* strains (multilocus sequence types [MLST] ST540 and ST2747, for which MICs of nitrofurantoin are 16 and 4 µg/ml, respectively) derived from stool samples from two Belgian outpatients with UTI. The strains were subjected to three stepwise platings on Mueller-Hinton agar supplemented with increasing nitrofurantoin concentrations (0.5- to 4-fold MIC for the parent strain). Whole-genome sequencing (Pacific Biosciences, Menlo Park, CA, USA) was done on the two parental and four nitrofurantoin-resistant strains generated under aerobic conditions (strains ST540-A and ST2747-A, with nitrofurantoin MICs of 256 and 128 µg/ml, respectively) and anaerobic conditions (strains ST540-AN and ST2747-AN, with nitrofurantoin MICs of 64 and 32 µg/ml, respectively). Genomic DNA was isolated with the MasterPure complete DNA and RNA purification kit (Epicentre, Madison, WI, USA), according to the manufacturer’s protocol. Library preparation and sequencing reactions were performed using the PacBio DNA template prep kit 2.0 (3 kb to 10 kb) and the PacBio DNA sequencing kit 2.0 with C2 chemistry. Sequence runs for six single-molecule real-time (SMRT) cells were performed on the PacBio RS II sequencer with a 1 × 180-minute movie time/SMRT cell. The SMRT Analysis portal version 2.0 was used for filtering the reads and subreads, with default parameters, and postfiltered data of ~320 Mb on each cell/per strain were considered for assembly. The six genomes were assembled using the Hierarchical Genome Assembly Process (HGAP), which is available with the SMRT Analysis packages and accessed through the SMRT Analysis Portal version 2.0. All six strains were sequenced with ~28-fold coverage spanning ~14 scaffolds for each strain. The scaffolds were ordered against an *in silico* whole-genome map of *E. coli* ATCC 8739 (GenBank accession no. CP000946) using the MapSolver software (Whole Genome Mapping; OpGen, Gaithersburg, MD) to derive a complete gapless chromosome (B. B. Xavier, J. Sabirova, P. Moons, J.-P. Hernalsteens, H. De Greve, H. Goossens, and S. Malhotra-Kumar, submitted for publication). The chromosome sizes of the ST540 strains are 4,758,628 bp, 4,807965 bp, and 4,875,674 bp. The ST2747 strains have chromosome sizes of 5,054,424 bp, 4,998,910 bp, and 5,090,326 bp, with a G+C content of ~51%. We used the Rapid Annotations using Subsystems Technology (RAST) server (6) and NCBI Prokaryotic Genome Automatic Annotation Pipeline (PGAAP) (7) for function-based annotation for all strains. The genomes of ST540 and ST2747 contain 4,587 and 4,859 protein-coding genes and 114 and 119 RNA genes, respectively, of which 89 encode tRNAs and 8 encode rRNAs. In addition, our genome annotation confirmed the duplication of the mobile elements and other single-nucleotide polymorphisms (SNPs) in ST540 and ST2747, which were identified using an inbuilt tool in the CLC Genomics Workbench 6.5.1 (CLC, Inc., Aarhus, Denmark).

### Nucleotide sequence accession numbers.

The genome sequences for all six strains have been deposited at DDBJ/EMBL/GenBank under the accession no. CP007265, CP007390, CP007391, CP007392, CP007393, and CP007394. The versions described in this paper are the first versions.

## References

[1] Manges AR Johnson JR Foxman B O’Bryan TT Fullerton KE Riley LW 2001 Widespread distribution of urinary tract infections caused by a multidrug-resistant *Escherichia coli* clonal group. N. Engl. J. Med. 345:1007–1013. 10.1056/NEJMoa011265 11586952

[2] McOsker CC Fitzpatrick PM 1994 Nitrofurantoin: mechanism of action and implications for resistance development in common uropathogens. J. Antimicrob. Chemother. 33(Suppl A):23–30. 10.1093/jac/33.suppl_A.23 7928834

[3] McCalla DR 1964 Effects of some nitrofurans on DNA synthesis and prophage induction. Can. J. Biochem. 42:1245–1247. 10.1139/o64-134 14228222

[4] Asnis RE 1957 The reduction of furacin by cell-free extracts of furacin-resistant and parent-susceptible strains of *Escherichia coli*. Arch. Biochem. Biophys. 66:208–216. 10.1016/0003-9861(57)90551-9 13395540

[5] McCalla DR Kaiser C Green MH 1978 Genetics of nitrofurazone resistance in *Escherichia coli*. J. Bacteriol. 133:10–16 338576 10.1128/jb.133.1.10-16.1978 PMC221970

[6] Aziz RK Bartels D Best AA DeJongh M Disz T Edwards RA Formsma K Gerdes S Glass EM Kubal M Meyer F Olsen GJ Olson R Osterman AL Overbeek RA McNeil LK Paarmann D Paczian T Parrello B Pusch GD Reich C Stevens R Vassieva O Vonstein V Wilke A Zagnitko O 2008 The RAST server: Rapid Annotations using Subsystems Technology. BMC Genomics 9:75. 10.1186/1471-2164-9-75 18261238 PMC2265698

[7] Angiuoli SV Gussman A Klimke W Cochrane G Field D Garrity G Kodira CD Kyrpides N Madupu R Markowitz V Tatusova T Thomson N White O 2008 Toward an online repository of Standard Operating Procedures (SOPs) for (meta)genomic annotation. OMICS 12:137–141. 10.1089/omi.2008.0017 18416670 PMC3196215

